# Laparoscopic combined resection of liver metastases and colorectal cancer: a multicenter, case-matched study using propensity scores

**DOI:** 10.1007/s00464-018-6371-1

**Published:** 2018-08-01

**Authors:** M. J. van der Poel, P. J. Tanis, H. A. Marsman, A. M. Rijken, E. C. Gertsen, S. Ovaere, M. F. Gerhards, M. G. Besselink, M. D’Hondt, P. D. Gobardhan

**Affiliations:** 1 Department of Surgery, Cancer Center Amsterdam, Amsterdam UMC, University of Amsterdam, Amsterdam, The Netherlands; 2 Department of Surgery, OLVG, Amsterdam, The Netherlands; 3 Department of Surgery, Amphia Hospital, Breda, The Netherlands; 4 Department of Digestive and Hepatobiliary/Pancreatic Surgery, Groeninge Hospital, Kortrijk, Belgium; 5 Department of Surgery, Cancer Center Amsterdam, Amsterdam UMC, University of Amsterdam, Room G4-146-1, Meibergdreef 9, P. O. Box 22660, 1100 DD Amsterdam, The Netherlands

**Keywords:** Colorectal liver metastases, Simultaneous, Liver resection, Colorectal resection, Laparoscopy

## Abstract

**Background:**

Combined laparoscopic resection of liver metastases and colorectal cancer (LLCR) may hold benefits for selected patients but could increase complication rates. Previous studies have compared LLCR with liver resection alone. Propensity score-matched studies comparing LLCR with laparoscopic colorectal cancer resection (LCR) alone have not been performed.

**Methods:**

A multicenter, case-matched study was performed comparing LLCR (2009–2016, 4 centers) with LCR alone (2009–2016, 2 centers). Patients were matched based on propensity scores in a 1:1 ratio. Propensity scores were calculated with the following preoperative variables: age, sex, ASA grade, neoadjuvant radiotherapy, type of colorectal resection and T and N stage of the primary tumor. Outcomes were compared using paired tests.

**Results:**

Out of 1020 LCR and 64 LLCR procedures, 122 (2 × 61) patients could be matched. All 61 laparoscopic liver resections were minor hepatectomies, mostly because of a solitary liver metastasis (*n* = 44, 69%) of small size (≤ 3 cm) (*n* = 50, 78%). LLCR was associated with a modest increase in operative time [206 (166–308) vs. 197 (148–231) min, *p* = 0.057] and blood loss [200 (100–700) vs. 75 (5–200) ml, *p* = 0.011]. The rate of Clavien–Dindo grade 3 or higher complications [9 (15%) vs. 13 (21%), *p* = 0.418], anastomotic leakage [5 (8%) vs. 4 (7%), *p* = 1.0], conversion rate [3 (5%) vs. 5 (8%), *p* = 0.687] and 30-day mortality [0 vs. 1 (2%), *p* = 1.0] did not differ between LLCR and LCR.

**Conclusion:**

In selected patients requiring minor hepatectomy, LLCR can be safely performed without increasing the risk of postoperative morbidity compared to LCR alone.

Approximately 15–25% of patients with colorectal cancer will have synchronous colorectal liver metastases at the time of diagnosis [1, 2]. Although it is clear that resection of both the colorectal primary tumor and the liver metastases offers the best chance for long-term survival, the optimal surgical strategy remains unknown. Randomized trials addressing the timing of both resections are lacking.

Traditionally, a staged resection is performed wherein a colorectal resection is followed by a hepatectomy at a later stage. In recent years, a ‘liver first approach’ is increasingly used, aimed at maximizing the change of completing the whole treatment plan [3, 4]. Surgery of the primary tumor first has the inherent risk of losing control of metastatic disease, especially considering the risk of severe complications such as anastomotic leakage delaying the hepatectomy. This is also the reason for an increasing role of induction therapy first, which allows for control of both the primary tumor and metastases. Simultaneous resection of both the primary tumor and liver metastases is an alternative approach in selected patients, either with or without induction therapy. However, some have argued that such a combined resection could lead to worse outcomes due to intestinal edema after hepatic pedicle clamping, transposition of colorectal bacteria to the liver transection surface, a decreased hepatic acute-phase response [5].

Despite these potential risks, many surgeons have stressed the benefits of a combined resection: shorter hospital stay and ‘one-stop’ treatment. Indeed, combined open liver and colorectal resection has been shown to be feasible and safe in selected patients [6–12]. Comparative studies on combined laparoscopic liver and colorectal resection (LLCR) are scarce. So far, the only comparative study of LLCR used a control group of minor liver resections [13]. This may not have been the most valid comparison, since laparoscopic colorectal cancer resection (LCR) typically carries more morbidity than a minor liver resection. To address the clinical concerns with LLCR we performed a multicenter case-matched study based on propensity scores, aiming to determine whether LLCR increases postoperative morbidity in comparison with LCR alone.

## Materials and methods

### Patients and design

This study reports the combined experience of three Dutch centers and one Belgian center with LLCR. All centers retrospectively reviewed their prospectively collected databases containing their complete experience with laparoscopic liver resection from 2006 until January 2017 (experiences ranging from 3 to 11 years) and selected all adults who underwent LLCR for colorectal cancer with synchronous liver metastases.

Data from the Dutch ColoRectal Audit (DCRA) between January 2009 and January 2017 from two participating centers were used to identify control patients. Similar data from the other two centers were unavailable. All adult patients undergoing LCR for colorectal cancer were included. Patients undergoing LLCR were matched with patients undergoing LCR alone based on propensity scores in a 1:1 ratio.

### Preoperative work-up

The primary tumor was diagnosed based on colonoscopy. Liver metastases were assessed with abdominal computed tomography (CT) scans with triphasic contrast enhancement and/or liver-specific double-contrast magnetic resonance imaging. To rule out extrahepatic disease, CT-chest and, in selected patients, positron emission tomography scans were used.

Prior to surgery, patients were discussed in a multidisciplinary team meeting attended by both liver and colorectal surgeons, gastroenterologists, medical oncologists, radiologists, radiotherapists and pathologists. Based on grading, size and location of the tumor (neo)adjuvant chemo- and/or radiotherapy regimens were considered according to national guidelines.

During work-up, a simultaneous resection was planned when both colorectal primary and liver metastases were considered resectable with curative intention, and the condition of the patient, judged by both the anesthesiologist and surgeon, was considered sufficient. Resectability was defined as the ability to achieve complete resection of the primary tumor as well as all metastases without the need for additional procedures, thus excluding patients with extrahepatic metastases. During the study period, patients requiring major liver resections and patients with liver lesions close to the portal pedicle or hepatic veins were not considered candidates for a simultaneous resection. Major liver resection was defined as any resection of 3 or more segments. Emergency colorectal resection because of bowel obstruction or perforation was also a contra-indication for LLCR. Simultaneous resections were usually performed by a single surgeon trained in both colorectal and liver surgery and discussed within the units liver surgery team. A decision regarding the surgical approach (laparoscopic or open) was made independently of the indication for surgery and was based on the patient’s performance status and location and size of both the primary tumor and metastases.

### Surgical technique

LLCR mostly started with the liver resection, thereby being able to decide on liver resection only in case a more extensive liver resection than planned based on preoperative imaging was required or more blood loss than expected. Laparoscopic liver resection was performed with the patient in supine position (or semiprone for liver resection of lesions in posterosuperior segments) and the surgeon in between the patient’s legs using three to four trocars in the upper abdomen. Laparoscopic ultrasound was used for detection of potentially occult lesions and to determine the plane of transection. Parenchymal transection was performed by using an ultrasonic dissection or bipolar sealing device alone or together with cavitron ultrasonic surgical aspirator (CUSA), with additional haemostasis using bipolar diathermy. Pedicle clamping during laparoscopic liver resection (Pringle manoeuver) was not standard practice. A laparoscopic 60-mm stapler was used to transect the portal pedicle and hepatic vein in case of a left lateral sectionectomy. Additional trocars were placed if necessary for laparoscopic colorectal surgery. A Pfannenstiel or vertical umbilical incision were mostly used for specimen extraction, followed by either an intra- or extracorporeal anastomosis.

### Outcomes

Baseline characteristics consisted of patient demographics, body mass index (BMI, kg/m^2^), American Society of Anesthesiology (ASA) grade, location of primary tumor (rectum, sigmoid, left colon, transverse colon or right colon), number, location and size of liver metastases on preoperative imaging, neoadjuvant treatment, type of resection of primary tumor, pathology of the primary tumor and the type and extent (minor/major) of liver resection.

Primary outcome was the rate of Clavien–Dindo grade 3 or higher complications including anastomotic leakage. The diagnosis of anastomotic leakage was based on clinical and radiological parameters, including any abscess occurring at the anastomosis, leakage of contrast fluid on imaging, endoscopically proven leakage or clinically suspect leakage requiring a reoperation. Other outcome parameters included operative time, intraoperative blood loss, need for conversion (to laparotomy, hand-assisted or hybrid technique), reason for conversion (e.g., adhesions, bleeding, inadequate access to the lesion, inadequate progress or other), need for a stoma, resection margins (*R*_0_ = tumor free, *R*_1_ = microscopic tumor involvement, *R*_2_ = macroscopic tumor involvement), pathology reported TNM stage of primary tumor, postoperative hospital stay, readmission (reason and timing) and 30-day mortality.

### Statistical analysis

Data analysis was performed using IBM SPSS Statistics for Windows version 24.0 (SPSS Inc., Chicago, IL, USA). Results were reported as median with interquartile range (IQR) as appropriate for continuous not normally distributed variables. If variables were normally distributed, results were reported as mean with standard deviation (SD). Categorical variables were reported as proportions. Propensity scores were calculated using a logistic regression model based on the following variables: age, sex, ASA grade, neoadjuvant radiotherapy, type of colorectal resection, T stage of primary tumor and N stage of primary tumor. Based on these propensity scores, LLCR were matched in a 1:1 ratio using a caliper of 0.1 to LCR alone. A Wilcoxon signed rank test was used to compare continuous, not normally distributed variables and ordinal categorical variables. Normally distributed continuous variables were compared using a paired T test. Finally, a McNemar test was used to compare binary and nominal categorical variables. A two-tailed *p* value of < 0.05 was considered statistically significant.

## Results

### Before matching

A total of 64 patients underwent LLCR between April 2009 and January 2017, which was a median of 3% (3–3.8) of the liver resections and 1% (1–2) of the colorectal resections performed per center during the study period. The mean annual number of LLCR per center was 4. Characteristics of liver metastases and resection are displayed in Table 1 and other patient characteristics are provided in Table 2. Most patients had minor comorbidities (ASA 1 and 2) (*n* = 51, 79%), a primary rectal/sigmoid tumor (*n* = 40, 63%) and a solitary liver metastasis (*n* = 44, 69%) of small size (≤ 3 cm) (*n* = 50, 78%).

**Table 1:** Liver metastases and resection characteristics

	Overall *N* = 64
Number of liver metastases	
1	44 (69)
2	9 (14)
3	4 (6)
> 3	7 (11)
Location of liver metastases	
Unilobar	52 (81)
Bilobar	12 (19)
Size of largest liver lesion, mm, median (IQR)	20 (13–30)
≤ 3 cm	50 (78)
> 3 cm	14 (22)
Surgical procedure	
Totally laparoscopic	56 (88)
Laparoscopic, hand-assisted	6 (9)
Laparoscopic, robot-assisted	2 (3)
Approach	
Liver first	43 (67)
Colon first	20 (31)
Liver resection strategy	
One stage resection only	54 (84)
One stage resection + RFA	1 (2)
Two stage resection without PVE	5 (8)
Two stage resection with PVE	4 (6)
Multiple liver resections	17 (27)
Type of liver resection	
Non-anatomical resection	45 (70)
Left lateral sectionectomy	7 (11)
Segmentectomy	12 (19)

All values in parenthesis are percentages unless mentioned otherwise. Percentages may not add up to 100 due to rounding. *IQR* inter-quartile range, *RFA* radiofrequency ablation, *PVE* portal vein embolization

**Table 2:** Baseline patient and tumor characteristics after matching based on propensity scores

	LLCR (*n* = 61)	LCR (*n* = 61)	*p*
Male sex	37 (61)	34 (56)	0.719
Age, mean (SD)	64 (11.6)	64 (13.1)	0.949
BMI, kg/m^2^, median (IQR)	25.8 (23.4–28.1)	25.2 (23.7–28.5)	0.958
ASA grade			0.988
ASA 1	15 (25)	14 (23)	
ASA 2	33 (54)	36 (59)	
ASA 3	12 (20)	9 (15)	
ASA 4	1 (2)	2 (3)	
Location primary			0.378
Rectum	12 (20)	18 (30)	
Sigmoid	27 (44)	23 (38)	
Left colon	4 (7)	4 (7)	
Transverse colon	0	2 (3)	
Right colon	18 (30)	14 (23)	
Neoadjuvant chemotherapy	12 (20)	5 (8)	0.039
Neoadjuvant radiotherapy	9 (15)	7 (12)	0.687
Type of resection primary			0.686
Low anterior resection/sigmoid resection	37 (61)	35 (57)	
Abdominoperineal resection	3 (5)	4 (7)	
Left colectomy	4 (7)	4 (7)	
Right colectomy	15 (25)	17 (28)	
Subtotal colectomy	2 (3)	1 (2)	
Pathology primary tumor			0.931
*T*_0_	2 (3)	0	
*T*_1_	2 (3)	2 (3)	
*T*_2_	3 (5)	8 (13)	
*T*_3_	46 (75)	42 (69)	
*T*_4_	8 (13)	9 (15)	
*N*+	48 (79)	46 (75)	

All values in parenthesis are percentages unless mentioned otherwise. Percentages may not add up to 100 due to rounding. *IQR* inter-quartile range, *BMI* body mass index, *ASA* American Society of Anesthesiology

All patients required minor liver resections: wedge metastasectomies [45 (70%)], left lateral sectionectomies [7 (11%)], and total segmentectomies [12 (19%)]. In 17 patients (25%), two or more resections were performed. For the primary tumor, low anterior/sigmoid resection (*n* = 38, 59%) was the most frequently performed procedure. Overall median operative time was 213 min (IQR 170–308) and blood loss was 200 ml (IQR 100–688). Conversion to laparotomy was necessary in three patients (5%), all due to inadequate access to the liver metastases. A Pringle maneuver was used in three patients (5%), of whom one developed an anastomotic leakage. Severe postoperative complications occurred in nine patients and included anastomotic leakage (*n* = 4), intra-abdominal fluid collections requiring radiological drainage (*n* = 2, one liver and one colon related), gastroparesis requiring endoscopic placement of a nasojejunal feeding tube (*n* = 1), wound bleeding requiring reoperation (*n* = 1) and cardiac arrhythmia requiring ICU admission (*n* = 1).

### After matching

A total of 1020 LCR were included in the study period and used for matching. After matching, 61 LLCR could be compared with 61 LCR. Baseline characteristics were comparable after matching based on propensity scores.

LLCR was associated with a 9-min longer operative time [206 (166–308) vs. 197 (148–231] min, *p* = 0.057) and 125 ml increase in blood loss [200 (100–700) vs. 75 (5–200) ml, *p* = 0.011]. Other operative outcomes did not differ between the groups. All outcomes after matching are displayed in Tables 2 and 3.

**Table 3:** Perioperative outcomes after matching based on propensity scores

	LLCR (*n* = 61)	LCR (*n* = 61)	*p*
Operative time, min, median (IQR)	206 (166–308)	197 (148–231)	0.057
Blood loss, ml, median (IQR)	200 (100–700)	75 (5–200)	0.011
Conversion	3 (5)	5 (8)	0.687
Peroperative incidents, Oslo classification			0.237
None	52 (85)	56 (92)	
Grade 1	6 (10)	4 (7)	
Grade 2	3 (5)	1 (2)	
Grade 3	0	0	
Stoma			0.317
None	51 (84)	46 (75)	
Double loop ileostomy	4 (7)	7 (12)	
End ileostomy	2 (3)	0	
End colostomy	4 (7)	8 (13)	
Severe complications	9 (15)	13 (21)	0.481
Anastomotic leakage	5 (8)	4 (7)	1.0
Postoperative stay, days, median (IQR)	6 (5–9)	7 (4–13)	0.164
Resection margins, *R*_0_	57 (93)	61 (100)	0.125
Readmission	7 (12)	8 (13)	1.0
30-day mortality	0	1 (2)	1.0

All values in parenthesis are percentages unless mentioned otherwise. Percentages may not add up to 100 due to rounding. *IQR* inter-quartile range

## Discussion

This first case-matched study using propensity scores to match LLCR in patients with synchronous colorectal cancer liver metastases with LCR alone found similar postoperative morbidity with a negligible increase in operative time (9 min) and blood loss (125 ml). Hospital stay was similar between LLCR and LCR alone, indicating a benefit of LLCR in these highly selected patients by omitting the need for a second hospital admission with its associated risks, costs and emotional burden for the patient. Based on these results, it seems worthwhile for experienced centers to screen and select patients with synchronous colorectal liver metastases who require minor hepatectomy for LLCR.

Despite single center reports on the feasibility and safety of LLCR, the true impact of adding a laparoscopic liver resection to a LCR on postoperative morbidity has never been investigated [14–17]. The potential benefits of a simultaneous resection in terms of patient satisfaction and reduction of costs seem obvious, but the concerns regarding raised postoperative morbidity are serious and should be addressed. This is also important since it has consistently been shown that there is no survival benefit of either one of the two strategies [12, 18]. Until now, the only comparative (non-matched) study included nine patients undergoing LLCR and 82 patients undergoing laparoscopic minor liver resection. Not surprisingly, giving the higher rate of complications after colorectal resection, morbidity was higher after LLCR versus a minor liver resection (22 vs. 1%) [13]. Other studies in open surgery have also reported unfavorable outcomes in terms of morbidity and even mortality when comparing a combined resection with liver resections only [19, 20]. A systematic review and meta-analysis, published in 2017, included 30 studies with a total of 2235 simultaneous and 3065 delayed open hepatectomies [12]. This study showed that a combined resection is feasible and can be performed without increasing postoperative morbidity compared to delayed hepatectomy. However, the results were clearly biased as patients in the delayed hepatectomy group more often had extensive liver lesions. The control group in these previous studies consisted of patients with only liver resections, instead of colorectal resections. This is somewhat surprising since the resection of the primary colorectal cancer is likely to dominate the risk of postoperative morbidity, rather than a minor liver resection. For instance, a large Dutch study demonstrated morbidity rates of 26 and 37% after laparoscopic and open colorectal cancer resections, respectively [21], whereas laparoscopic minor and open minor liver resections are associated with morbidity rates of 13 and 30%, respectively [22]. Furthermore, the main concerns with LLCR focus on adding morbidity to the colorectal resection due to congestion and added intraoperative fluid load potentially leading to increased rate of anastomotic leakage and septic complications.

Laparoscopy could have played a role in the relatively low rate of major morbidity after LLCR in this series. In most centers nowadays, laparoscopic surgery is considered standard of care for primary resectable colorectal cancer, and most recent consensus meetings on laparoscopic liver surgery have declared laparoscopy the standard for minor liver resections as well [23–25]. In both procedures, a laparoscopic approach has been associated with faster recovery and shorter postoperative hospital stay, as well as decreased complication rates [21, 22, 26, 27]. Furthermore, the decreased need for pedicle clamping during laparoscopic liver surgery related to the intra-abdominal pressure during laparoscopy could decrease the risk of additional morbidity during LLCR [28–32]. One meta-analysis of three studies comparing LLCR with open combined colorectal and liver resections reported shorter hospital stay after LLCR, without compromising safety [33].

The current study had several limitations. First, the retrospective design clearly introduced a risk of selection bias. Selection criteria were, however, essentially similar in the four participating centers and are described in the methods section. Even though laparoscopic major liver resections were performed in all centers during the study period, these patients were not considered to be candidates for LLCR. Second, the size of the cohort did not allow for identification of subgroups, for instance comparing outcome after left- and right-sided colon cancer resection. A randomized controlled trial designed to answer the question whether LLCR is superior to a staged resection seems unlikely, so matching based on propensity scores is probably the next best methodology. Larger cohorts could help to further identify subgroups when it comes to the surgical treatment of synchronous colorectal liver metastases. In order to increase the potential of finding a matching LCR control patient, metastasized colorectal tumors were not excluded. This means that some of these patients might undergo further surgery for metastatic disease after colorectal resection. The aim of this study, however, was to investigate short-term outcomes and there is no current literature available suggesting that in situ metastatic disease influences the outcomes of colorectal or liver surgery. Long-term results of simultaneous resections, such as disease-free and overall survival, remain uncertain, especially considering the possible extravasation of tumor cells during colorectal resection that could settle down in areas of tissue damage and inflammation such as the liver surface after resection. Finally, the potential advantages of LLCR were not specifically addressed in this study, which can be seen as a limitation. The most accurate comparison would be between LLRC and the cumulative of both colorectal and liver resection in a delayed setting. These patients were difficult to identify and due to the selection bias for the extent of liver resection matching would not have been possible.

Postoperative hospital stay has consistently been shown to be decreased with open combined resection when compared to the cumulative length of stay when performing sequential resections [12]. The current study did not show a significant difference in length of postoperative stay between the groups. However, as the delayed liver resection was not taken into account in this study, this outcome would favor LLCR as all hospitalization for the delayed liver resection can be avoided. The same applies to operative time and intraoperative blood loss. It is interesting to note that the operative time in LLCR was only 9 min longer on average than in the laparoscopic colorectal resection group. This seems unlikely, but abdominal access and closure of extraction site and trocar ports had to be performed only once in case of LLCR if compared to a staged procedure, which saves a lot of time. Not all centers may have surgeons skilled in both laparoscopic colorectal and liver surgery. This may not be a major problem but does require close communication on the details of patient selection, patient positioning, and trocar placement. On the other hand, centralization of these specific cases for simultaneous resection to experienced centers is probably better. Finally, patient satisfaction is impossible to measure in a retrospective setting but it would seem unlikely that patients would favor staged operations over LLCR.

In conclusion, this study showed that LLCR is feasible and does not increase postoperative morbidity compared to LCR alone, in selected patients with synchronous colorectal liver metastases requiring a minor liver resection, operated in experienced centers.
